# Uncertainty quantification of granular computing-neural network model for prediction of pollutant longitudinal dispersion coefficient in aquatic streams

**DOI:** 10.1038/s41598-022-08417-4

**Published:** 2022-03-17

**Authors:** Behzad Ghiasi, Roohollah Noori, Hossein Sheikhian, Amin Zeynolabedin, Yuanbin Sun, Changhyun Jun, Mohamed Hamouda, Sayed M. Bateni, Soroush Abolfathi

**Affiliations:** 1 School of Environment, College of Engineering, University of Tehran, Tehran, 1417853111 Iran; 2 Faculty of Governance, University of Tehran, Tehran, 1439814151 Iran; 3 Department of Geospatial Information Systems, College of Engineering, University of Tehran, Tehran, 1439957131 Iran; 4 School of Civil Engineering, College of Engineering, University of Tehran, Tehran, 1417613131 Iran; 5 College of Hydrology and Water Resources, Hohai University, Nanjing, 210098 China; 6 Department of Civil and Environmental Engineering, College of Engineering, Chung-Ang University, Seoul, 06974 Korea; 7 Civil and Environmental Engineering and the National Water Center, United Arab Emirates University, Al Ain, 15551 Abu Dhabi United Arab Emirates; 8 Department of Civil and Environmental Engineering and Water Resources Research Center, University of Hawaii at Manoa, Honolulu, HI 96822 USA; 9 School of Engineering, University of Warwick, Coventry, CV4 7AL UK

**Keywords:** Environmental sciences, Hydrology

## Abstract

Discharge of pollution loads into natural water systems remains a global challenge that threatens water and food supply, as well as endangering ecosystem services. Natural rehabilitation of contaminated streams is mainly influenced by the longitudinal dispersion coefficient, or the rate of longitudinal dispersion (*D*_*x*_), a key parameter with large spatiotemporal fluctuations that characterizes pollution transport. The large uncertainty in estimation of *D*_*x*_ in streams limits the water quality assessment in natural streams and design of water quality enhancement strategies. This study develops an artificial intelligence-based predictive model, coupling granular computing and neural network models (GrC-ANN) to provide robust estimation of *D*_*x*_ and its uncertainty for a range of flow-geometric conditions with high spatiotemporal variability. Uncertainty analysis of *D*_*x*_ estimated from the proposed GrC-ANN model was performed by alteration of the training data used to tune the model. Modified bootstrap method was employed to generate different training patterns through resampling from a global database of tracer experiments in streams with 503 datapoints. Comparison between the *D*_*x*_ values estimated by GrC-ANN to those determined from tracer measurements shows the appropriateness and robustness of the proposed method in determining the rate of longitudinal dispersion. The GrC-ANN model with the narrowest bandwidth of estimated uncertainty (bandwidth-*factor* = 0.56) that brackets the highest percentage of true *D*_*x*_ data (i.e., 100%) is the best model to compute *D*_*x*_ in streams. Considering the significant inherent uncertainty reported in the previous *D*_*x*_ models, the GrC-ANN model developed in this study is shown to have a robust performance for evaluating pollutant mixing (*D*_*x*_) in turbulent environmental flow systems.

## Introduction

Discharge of pollution loads into streams threatens the water and food supply, along with aquatic biodiversity at a global scale^1,2^. Natural rehabilitation of polluted streams is mainly characterized by the longitudinal dispersion coefficient, or the rate of longitudinal dispersion (*D*_*x*_ or *K*_*x*_), a key parameter in river water quality models with large temporal and spatial variations. A challenging task in the study of the pollutant fate and transport in turbulent flow systems (e.g., streams) is determining *D*_*x*_ for numerical and analytical water quality models^3,4^. *D*_*x*_ is the most predominant factor influencing the pollutant concentration at the downstream of the point of accidental pollution^5–8^. Starting from the late 1960s, the mechanism of *D*_*x*_ determination in streams was introduced by Fischer^9^. Fischer^10^ proposed an analytical formula to estimate *D*_*x*_ that required detailed knowledge of the flow-geometric conditions of the system under study.

Given that the flow-geometric data for streams, especially in large meandrous channels, are highly variable in temporal and spatial scales, such data are not readily measured and available. Also, the complex numerical procedures required to solve Fischer^10^ equation, have led to introduction of several simplifications to determine *D*_*x*_. Hence, the estimations of *D*_*x*_ from the simplified models can largely deviate from the field-based estimated measurements^11,12^. These simplifications are mainly exclusion of some variables which are difficult to access such as flow-geometric irregularities that influence dispersion mechanism in streams. Although in many cases the impact of the excluded variables is somewhat embedded in other variables used in *D*_*x*_ estimation models, they do not fully represent the complex interactions between the absent variables and *D*_*x*_. For example, friction term (i.e., rate of flow velocity to shear velocity − U/U^*^), as a readily accessible input for *D*_*x*_ estimation models, to some extend can represent the impact of lateral and vertical irregularities in streams that affect the rate of dispersion^13^. However, these irregularities produce shear flows and secondary currents that can alternate the *D*_*x*_. Simultaneously, the former causes an increase in *D*_*x*_ whilst the latter decreases *D*_*x*_^14–18^. The complex interactions between the flow-geometric data and dispersion mechanism prohibit reaching an accurate estimation of *D*_*x*_ in streams whilst some effective variables on dispersion mechanism are excluded (e.g., stream bed shape factor and sinuosity).

In recent decades, and with the advancement in artificial intelligence (AI) models, they became powerful tools to solve complex engineering problems^19–27^. A number of AI-based studies have been conducted to enhance the accuracy of *D*_*x*_ estimation in turbulent flow systems such as natural streams^28–31^. Given that AI techniques are able to map the complex non-linear input–output relationships even when some important information is missing^32^, their applications in estimating the *D*_*x*_ have been investigated by several studies^28–31,33–43^. However, complex nature of dispersion mechanism in turbulent flow systems with variations in both spatial and temporal scales, as well as the inevitable simplification assumptions that are needed for the modelling will result in uncertainty of *D*_*x*_ estimation using AI-based models. The uncertainty in the output of hydrological models is largely resulted by factors such as input-data uncertainty, model uncertainty and parameter uncertainty^44–51^. Intensive efforts have been made to investigate the uncertainty of physics-based hydrological models, which led to good understanding of the different sources of uncertainty and their quantification approaches in hydrological models^44–51^. However, there still remains a significant need to understand and quantify the uncertainty associated with AI-based hydrological models, especially for water quality modelling. In river water quality modelling, the majority of existing AI-based studies are conducted to find the best point estimation, without much attention towards the uncertainty quantification of the model predictions. AI-based models, as data-driven techniques, have not been elaborated to consider the physical mechanisms of the objective parameter under study. In contrast with the physics-based models, AI-based models discover and learn the underlying physical mechanisms that govern water quality parameters using a training process^38,41,42^. The performance of training procedure depends on the sampling patterns selected to tune the AI-based model. Therefore, given that the predictions of AI-based models are highly impacted by the data used for training, any changes in the selected training data can impose large uncertainty in the model output. In a study conducted by Noori et al.^42^, they reported that although the AI techniques outperform empirical-based models for estimation of *D*_*x*_, their predictions are still subject to uncertainty induced by changes in their training patterns. The inaccuracy in estimation of the *D*_*x*_ using AI models can limit water quality assessment and design of appropriate measures to improve the water quality of aquatic flows. Hence, developing a robust methodological framework to quantify the prediction uncertainty of the *D*_*x*_ from AI-based models is essential for developing appropriate AI-based water quality models.

Granular computing (GrC) model is a highly efficient AI-based model which has recently shown an excellent potential to solve complex engineering problems^52–56^. GrC model is a novel tool capable of applying the granules in the process of nonlinear problem solving^52^. In the GrC model, the natural rules between the data are extracted by means of the rule mining algorithm, operating on a set of information arranged as information table. The granule measures involved in the process of information mining, has made GrC as a powerful tool to map a set of inputs to a set of outputs in different fields of science and engineering^52,53^. However, similar to other AI models, the GrC performance can be adversely influenced by the selection of training patterns. Therefore, the effects of changes in training patterns on the performance of GrC model should be investigated, to understand and quantify the degree of uncertainty in the model’s prediction of *D*_*x*_ in water quality assessments. Previous studies which examined the application of GrC model for *D*_*x*_ estimation in natural streams did not investigate the prediction uncertainty introduced by the model training patterns^39,43^. In this study, we first coupled an artificial neural network (ANN) with rules information in the GrC (GrC-ANN) to improve the GrC model’s performance. Encoding the given information used in the GrC into a feed forward multi-layer structure, i.e. ANN, enhances the GrC model to use all information available in the dataset to decide about different presented patterns. Then, a *D*_*x*_ predictive model was developed using GrC-ANN modelling technique. Finally, a comprehensive uncertainty analysis method was proposed to compare the accuracy of *D*_*x*_ predicted by the GrC-ANN with other AI-based *D*_*x*_ models in the literature. Our proposed method quantifies the GrC-ANN prediction’s uncertainty based on the model response to change in the selected training patterns using a modified bootstrap method^12^.

## Methods

### Longitudinal dispersion

**Figure 1:**
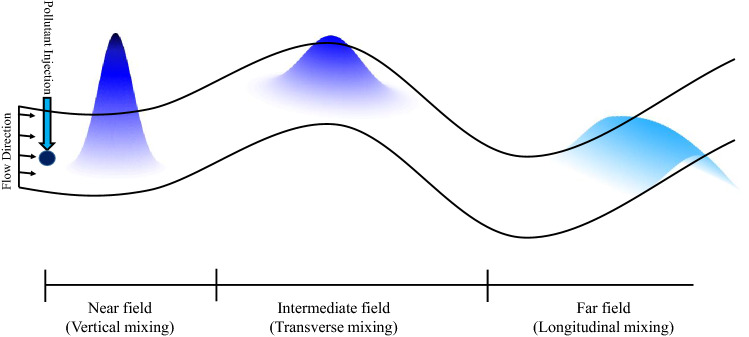
Schematic of concentration profiles and pollutant mixing in streams. (adapted from Kilpatrick and Wilson^59^).

Non-reactive pollutant mixing in aquatic systems is a complex three-dimensional (3-D) flow process, consists of molecular and turbulent diffusion, and shear dispersion (referred to as “dispersion”) mechanisms. The dispersion is the net trace of velocity shear over the flow width and depth, and the turbulent mixing^11^. In the natural streams, which are specifically much longer than width or depth of the flow, the pollutants become well-mixed in the vertical and transverse directions rather than the longitudinal mixing (Fig. 1). Therefore, pollutant fate and transport in streams is usually studied by the application of 1-D mixing model quantified by the advection–dispersion equation as follows^57,58^:

1 $$\frac{\partial C}{\partial t}+U\frac{\partial C}{\partial x}={D}_{x}\frac{{\partial }^{2}C}{\partial {x}^{2}}.$$

In Eq. (1), *C* and *U*, are the averaged cross-sectional concentration and averaged longitudinal velocity, respectively, *t* denotes time and *x* is the longitudinal coordinate in the stream-wise direction.

Ideally, vertical and (transverse) dispersion in streams takes place close to (in intermediate fields from) the pollutant discharge location, whilst the longitudinal dispersion occurs far from the pollutant discharge point, where solute become readily well-mixed in both vertical and transverse directions (Fig. 1). In streams, the longitudinal dispersion usually varies form 10^–1^ to 10^7^ m^2^/s^10,13,60,61^ and the diffusion coefficient ranges from 10^–9^ (molecular) to 10^–2^ m^2^/s (turbulent)^5^. Therefore, dispersion is the dominant mechanism of mixing process, by several orders of magnitude^62^, highlighting the necessity of developing robust methodological approach to quantify the dispersion and mixing coefficient in the streams.

### ***D***_***x***_ parametrization

Pioneering work on quantification of dispersion mechanism in pipes date back to Taylor’s studies^63,64^. Thereafter, Taylor’s approach was used for quantifying dispersion in streams with the assumption of no limits for the width of the channel by Elder^65^. However, the Elder’s formula underestimates the dispersion in natural streams, as it does not consider the influence of the lateral velocity shear^10,66^. In streams, the lateral velocity shear mechanism plays a more dominant role in determining the mixing compared to the vertical shear. On this basis, Fischer^9^ derived an analytical formula for determining *D*_*x*_ as:

2 $$D_{x} = \left\{ {\int\limits_{0}^{W} {h\left( y \right)\mathop {u}\limits^{\prime } \left( y \right)} \int\limits_{0}^{y} {\left[ {1/\varepsilon _{t} \left( y \right)h\left( y \right)} \right]} \int\limits_{0}^{y} {h\left( y \right)\mathop {u}\limits^{\prime } \left( y \right)dydydy} } \right\}/A,$$

where, *W* denotes the local flow width, *x* is the longitudinal coordinate, *y* is the lateral coordinate, $\mathop u\limits^{{\prime }} \left( y \right)$. is the local velocity deviation, $h\left(y\right)$ represents the local flow depth, ${\varepsilon }_{t}\left(y\right)$ is local lateral mixing coefficient, and *A* represents the local flow cross-sectional area.

In Eq. (2), the flow is supposed to be 1-D, i.e., the pollutant is well-mixed in both vertical and lateral directions, a condition that is rarely satisfied in turbulent flow systems such as large meandrous streams and even in laboratory flumes, due to existence of secondary currents^67^. Fischer^9^ equation has been derived based on the assumption that the dispersion is controlled by lateral shear rather than the vertical shear, a condition that may not be well-satisfied for the narrow and deep rivers where the aspect ratio (i.e., river flow width to depth − *W/H*) is small^5^. These drawbacks of Eq. (2) lead to inaccurate estimation of *D*_*x*_ compared to those values determined from tracer measurements. The deviation between *D*_*x*_ values estimated by Eq. (2) and those true values is maximum for the case of non-uniform flow in real meandrous streams, albeit Fischer^9^ model can well approximate the dispersion for the case of uniform flows^68^. In addition to the inherent drawbacks in practical application of the Eq. (2), it also requires detailed information on the geometrical properties (i.e. cross-section, bathymetry) of stream, as well as the lateral flow velocity profiles. Collecting such information is rather costly and time consuming, and often requires very detailed flow measurements which are not readily available. Therefore, practical application of Fischer^9^ model is limited.

To address the difficulties in using Eq. (2), Fischer^69^ suggested a simplified empirical equation that correlates *D*_*x*_ with pertinent dimensionless variables of *W/H* and U/U^*^ as follows:

3 $$\frac{{D}_{x}}{H{U}^{*}}=a{\left(\frac{W}{H}\right)}^{b}{\left(\frac{U}{{U}^{*}}\right)}^{c}.$$

Fischer^69^ modified empirical formula for determining the dispersion coefficient (Eq. 3), has been widely used and validated by other researchers^11–13,28–31,70^ and rely on the parameters which can be practically determined for natural streams.

### Data collection

This study aims to estimate *D*_*x*_ in streams using GrC-ANN model. In this regard, a global tracer database consisting of 503 observations from natural streams and laboratory flumes was used to develop the model and validate the performance of the proposed GrC-ANN model. This database was compiled by Riahi-Madvar et al.^71^, and include data on the friction term, aspect ratio, and with *D*_*x*_ ranging between ~ 0.00 to ~ 1800 m^2^/s. Although the database used in this study is more comprehensive compared to other studies on *D*_*x*_ estimation, it does not fully include extreme high values of *D*_*x*_^12^. The reported *D*_*x*_ values in the literature are within the range of near to zero (in the laboratory flumes) to extreme high value of 6800 m^2^/s in large and irregular-shaped rivers^72^. The maximum *D*_*x*_ used in this study is ~ 1800 m^2^/s, which is related to dispersion in natural streams with irregular hydraulic-geometric characteristics, and dispersion values greater than what is used in this study are extremely rare in environmental hydraulics problems. Therefore, the extremely high *D*_*x*_ values were excluded from the database as outliers, given that they significantly impact the statistical analyses^12^. However, ${D}_{x}/H{U}^{*}$ parameter in the database used here has a non-normal distribution as described by Noori et al.^12^. Using a preliminary investigation, it was found that no significant difference exists between the GrC-ANN model outputs with the normalized and raw ${D}_{x}/H{U}^{*}$ data. Therefore, the raw data was considered for further investigations in this study.

### GrC-ANN model development

In-depth description of the ANN, GrC and GrC-ANN approaches for *D*_*x*_ modelling in streams and the model development procedures are given in Noori et al.^39^ and Ghiasi et al.^43^, respectively. Further detailed information about these models documented in the literature^52–55,73–75^. Hence, we shortened the descriptions of GrC-ANN model developed in this study.

**Figure 2:**
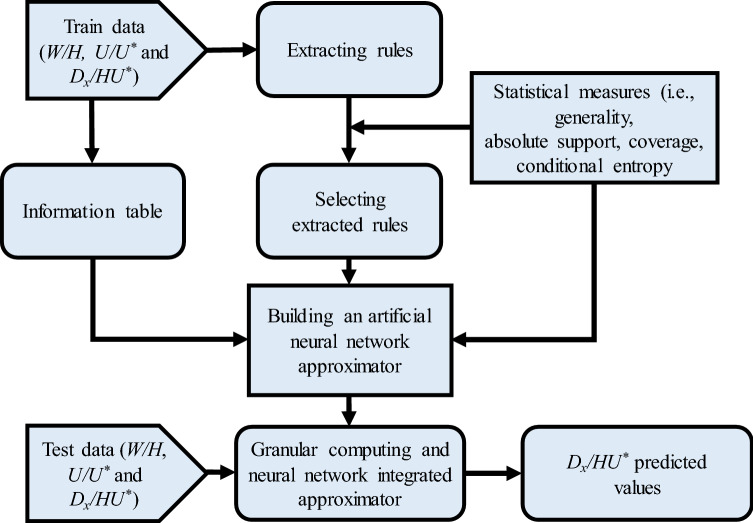
Procedure of integrating GrC and ANN for determining longitudinal dispersion coefficient.

- **GrC model**
  Granular Computing models are superset of the rough set theory, interval computations and the theory of fuzzy information granulation^52^. GrC model is a data processing method based on multiple levels of data granularity. In this method, the whole dataset is divided into granules and clusters (or subsets), which categorizes individual elements of the whole dataset based on the existing similarity between objects to put them in different granules. Then, a set of rules is extracted over concepts *ϕ* and *Ψ* in the form of IF–THEN: “If an objective satisfies *ϕ*, THEN the object satisfies *Ψ*”. Here, concepts *ϕ* and *Ψ* are a set of attribute-values for a set of objects and the assigned output value, respectively. In the process of rule extraction, GrC algorithm forms all the possible granules to extract every relation between the patterns, i.e. extracted rules, regardless of their importance or accuracy. Following rules extraction procedure, the algorithm applies statistical measures on granules formed in order to select the best set of possible rules, i.e. pruned rules, to form the regression rule set^52–55^.
  Generality (*G*), absolute support (*AS*), coverage (*CV*), and conditional entropy (*CE*) are the statistical measures used by the GrC to extract the rules. The generality of concept *ϕ*, i.e. *G*(*ϕ*), displays the relative size of constructive granule of this concept, defined by Eq. (4)^76^:
  4 $$G\left(\phi \right)=\frac{\left|m\left(\phi \right)\right|}{\left|U\right|},$$
  where $\left|m\left(\phi \right)\right|$ is the size of the granule and $\left|U\right|$ is the size of the entire domain. *G*(*ϕ*) varies between 0 and 1. Higher values of generality describe the rule as a more common concept, which is more probable to occur. On the other hand, high *G*(*ϕ*) can bias the model towards the patterns observed during the training process.
  *AS*, as the conditional probability in the case that a randomly selected object satisfies both *ϕ* and *Ψ*, can be obtained from Eq. (5) and describes the strength of a rule in assigning similar outputs to a set of input values^73^. *AS* = 1, if and only if $m\left(\phi \right)$ ⊆ $m\left(\Psi \right)$.
  5 $$AS\left( {\phi \to \Psi } \right) = \frac{{\left| {m\left( {\phi \Lambda \Psi } \right)} \right|}}{{\left| {m\left( \phi \right)} \right|}}.$$
  *CE*, represented by $H\left(\Psi \mid \phi \right)$, reveals the uncertainty of formula *ϕ* based on formula *Ψ* and is defined by Eq. (6)^73^. *CE* ensures the model reliability and robustness, by filtering out the rules that are providing information which is not supported by other rules in the rule set, even if these rules have misleading acceptable values for other statistical measures.
  6 $$H\left(\Psi \mid \phi \right)=-\sum_{i=1}^{n}p\left({\Psi }_{i}\mid \phi \right)\mathrm{log}\left(p\left({\Psi }_{i}\mid \phi \right)\right),$$
  where, $$p\left({\Psi }_{i}\mid \phi \right)=\left|m\left(\phi \cap {\Psi }_{i}\right)\right|/\left|m\left(\Psi \right)\right|$$.
  *CV* denotes the conditional probability of a randomly selected object to satisfy *ϕ*, while satisfying *Ψ*^73^. This parameter shows the strength of a rule in predicting accurate output values if different training patterns are provided to the model.
  7 $$CV\left(\phi \to \Psi \right)=\frac{\left|m\left(\phi \cap \Psi \right)\right|}{\left|m\left(\Psi \right)\right|}.$$
  In this study, GrC extracts the rules from the global tracer database consisting of 503 observations from natural streams and laboratory flumes based on the *CE* and *AS* statistical measures, so that the rules with the minimum *CE* value and the maximum *AS* are extracted from the database. To form a granular decision tree, the priority of rules in the tree is determined based on the *G* and *CV*.
- **ANN model**
  An ANN consists of a set of neurons, as the smallest computational units of the model, organized in different layers joint by connection weights. The first and last layers are the input and output layers of the network, respectively. The layers in the middle of the network are hidden and contain computing neurons. To construct an ANN for a predictive modelling purpose, training data are introduced to the network. Then, the network starts the learning process by determining connection weights and biases based upon the resulting error at the output nodes^77^. Upon obtaining the connection weights and biases, the network is ready to do a classification or regression task.
- **GrC-ANN model**
  A basic GrC model has two major deficiencies. First, it prioritizes rules based on their obtained parameters and uses the first rule satisfied by the input data to define its output. Second, it cannot make use of information provided in the rule set and makes a prediction by only using one rule^73–75^. Hence, to compensate for these deficiencies, the GrC-ANN model proposed in this study uses an integration of GrC rule generation algorithm and ANN model (Fig. 2). The GrC-ANN approach allows the model to use the mentioned rule quality parameters (i.e. *G*, *AS*, *CV*, and *CE*) to construct the approximator structure, instead of common time-consuming iterative learning procedure used by ANN model^48^. Given the input patterns, the GrC-ANN model tunes the network by re-forming the granules and applying statistical measurements performed by the GrC approach. Re-forming the granules also re-forms the rules, which results in different number of rules and different statistical measurements. *CE* plays an important role in tuning the model. Keeping *CE* close to zero filters out inconsistent rules by removing them or giving them less importance. GrC-ANN tries to minimize the number of rules by minimization of *CE* and maximization of *G*, *AS* and *CV*^52,53^.

**Figure 3:**
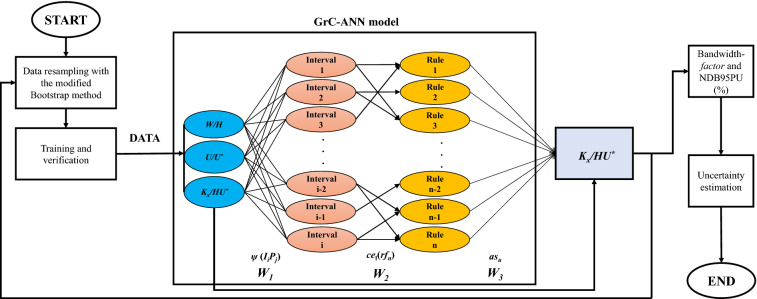
Schematic of the GrC-ANN model structure developed for this study.

The GrC-ANN structure proposed in this paper, similar to the conventional neural networks, comprises of layers including the input layer, two computing layers, and the output (aggregation) layer (Fig. 3). The layers within the proposed GrC-ANN model are customized to ensure robust predictions of *D*_*x*_. The number of nodes in the input layer are set equal to attributes of the data records (i.e., *W/H*, U/U^*^, and ${D}_{x}/H{U}^{*}$). Computing layers are comprised of two inner-connected layers including pattern layer and rule firing layer. The computing layers receive values that are valid according to the criteria determined in the input layer. Computing layers’ characteristics are fully data driven. Pattern layer nodes act as transformers, normalizing quantized valid values of the criteria in the input layer as the rule firing nodes expect. Rule firing nodes use the data provided from the measured and selected rules to aggregate the received values, turning them into predictions. The third layer contains the set of qualified extracted rules by GrC-ANN and embeds the classification rules. The aggregation layer assigns an output value to the input pattern of the data. The connection weights of the rule-firing layer and the aggregation layer are given by the statistical measure of absolute support provided by the corresponding rule to its output value, to consider the accuracy of the rules in determining that output value^43,52,53^.

The proposed GrC-ANN approach benefits from some advantages that are absent in both GrC and ANN models. Utilizing tangible information obtained from the rule measures in the form of neurons, layers and connection weights improve the transparency of the constructed model^43^. Since the given information are encoded in a feed forward multi-layer structure, similar to ANNs, the GrC-ANN will be able to use all information available in the dataset to decide about each presented data pattern, which is an improvement to rule-based classifiers, such as GrC^52^. Replacing the learning part of an ANN with the information from rule quality measures ensures that no connections or nodes are remained without a transparent description. This is an improvement to the conventional ANNs which contain hidden neurons and obtain their connection weights and biases by learning through a black-box learning algorithm^43^. A conventional ANN provides results which is influenced by initial weights generated in a random manner, yielding to different results from the same set of training information, lacking the ability to describe them. GrC-ANN provides a robust network and can be manipulated by defining rule measure thresholds. Overall, these advantages improve the accuracy of the proposed GrC-ANN predictions compared to conventional GrC and ANN models^43,52^. Although GrC-ANN model reduces the computational time needed for model training by removing the learning procedure in the ANN model, it requires more computational cost than ANN model due to the procedure of extracting high-quality classification rules. In general, the computational cost for the GrC-ANN model is in the order of: $\mathrm{O}\left({n}^{2}\times {p}_{1}\times a\times m+\times {p}_{2}\times n\times a\times \left(l\times r\right)\right)$, aggregating training and verification time, where *n* is the number of iterations, *a* is the number of attributes for the patterns, *m* is the number of GrC measure parameters, *r* denotes the number of rules used in prediction, *l* is the number of layers in the network, *p*_1_ and *p*_2_ are the number of input patterns for training and verification, respectively^53,73–75^.

### Uncertainty quantification

Similar to other data-driven models, the GrC-ANN model minimizes the error function based on the data fed with the aid of a supervised algorithm throughout the training process^43^. Hence, model training plays a vital role in quantification of the GrC-ANN model’s uncertainty caused by different tuning sets. In this study, the GrC-ANN model was tuned to map the input parameters, i.e. *W/H* and U/U^*^, to the target ${D}_{x}/H{U}^{*}$, based on finite training patterns resampled from 503 observations of the global tracer database. Probabilistically, each training pattern used for tuning the GrC-ANN model is different from others resampled from the global database. Thus, each training pattern could produce different set of GrC-ANN parameters, and predictive outputs for the estimation of ${D}_{x}/H{U}^{*}$.

The modified bootstrap method suggested by Noori et al.^12^ was used to resample distinct training patterns for tuning the GrC-ANN model for ${D}_{x}/H{U}^{*}$ predictions. This method ensures that the chosen training patterns are fully representative of the statistical characteristics of the 503 tracer experiments of the global database used in this study. This is particularly important since the global database used in this study rarely has large *D*_*x*_ instances^12^, denoting that these large dispersion values are likely to be under-represented in the training patterns chosen by the conventional bootstrap technique. This issue can result in poor training of the GrC-ANN model and consequently increases the model’s uncertainty in prediction of ${D}_{x}/H{U}^{*}$. Detailed description of the bootstrap method is given by Efron and Tibshirani^78^, while the modified the bootstrap method adopted in this study is described by Noori et al.^12^.

Different outputs of the ${D}_{x}/H{U}^{*}$ GrC-ANN model in the verification stage, i.e. due to the change in the training patterns, were used as a measure of the model’s uncertainty^79^. An interval band of the GrC-ANN estimations of ${D}_{x}/H{U}^{*}$ was computed, with a level of significance of 95%. Then, two measures were introduced to assess the ${D}_{x}/H{U}^{*}$ prediction variations in the different responses of the GrC-ANN model in verification stage including bandwidth-*factor* and the number of bracketed ${D}_{x}/H{U}^{*}$ data using 95% of predicted uncertainties (NBD95PU) as shown in Eqs. (8) and (9), respectively^80^. Given these two measures, the uncertainty in estimation of the ${D}_{x}/H{U}^{*}$ GrC-ANN model in verification stage was quantified.

8 $${\text{bandwidth}} {-} factor = \left\{ {\left( {\frac{1}{n}} \right)\sum\limits_{{i = 1}}^{n} {\left( {X_{U} - X_{L} } \right)} } \right\}/\sigma _{x} ,$$

9 $$\mathrm{NBD}95\mathrm{PU }\left(\mathrm{\%}\right)=\left(1/n\right)\mathrm{count}\left\{Q\left|\left({X}_{L}\le Q\le {X}_{U}\right)\right.\right\},$$

where ${\sigma }_{x}$ is the standard deviation of the target ${D}_{x}/H{U}^{*}$, and ${X}_{U}$ and ${X}_{L}$ are the maximum and minimum of the estimated ${D}_{x}/H{U}^{*}$ for each training pattern, respectively.

Figure 3 illustrates a detailed description of the model development and uncertainty quantification process proposed for this study.

## Results and discussion

### Tuned GrC-ANN models

**Figure 4:**
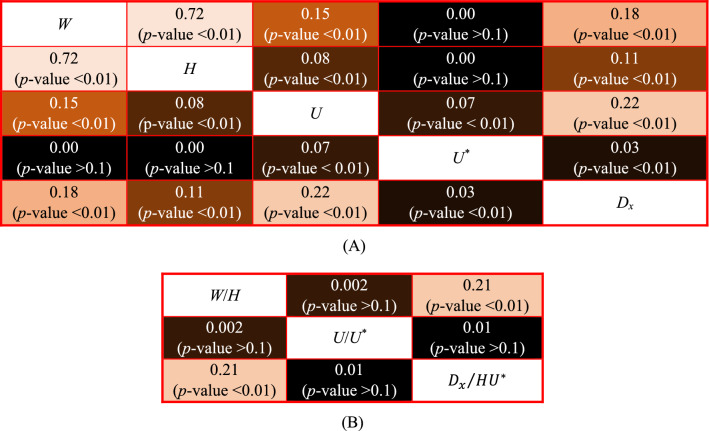
The correlation coefficient plots of (**A**) *W*, *H*, *U*, U^*^, and *D*_*x*_, (**B**) *W/H*, U/U^*^ and ${D}_{x}/H{U}^{*}$.

The correlation amongst the input parameters, i.e. *W*, *H*, *U*, U^*^, and *D*_*x*_ is shown in Fig. 4A. The correlation coefficients for the model variables in dimensionless format, i.e. *W/H*, U/U^*^ and ${D}_{x}/H{U}^{*}$ and the corresponding statistical significance level are illustrated in Fig. 4B. In dimensional form, ${D}_{x}/H{U}^{*}$ is more correlated with the geometrical configuration *W/H* of the stream (correlation coefficient = 0.21, *p*-value < 0.1) than the flow characteristic U/U^*^ (correlation coefficient = 0.002, *p*-value > 0.1), confirming the results reported by Noori et al.^12^.

**Figure 5:**
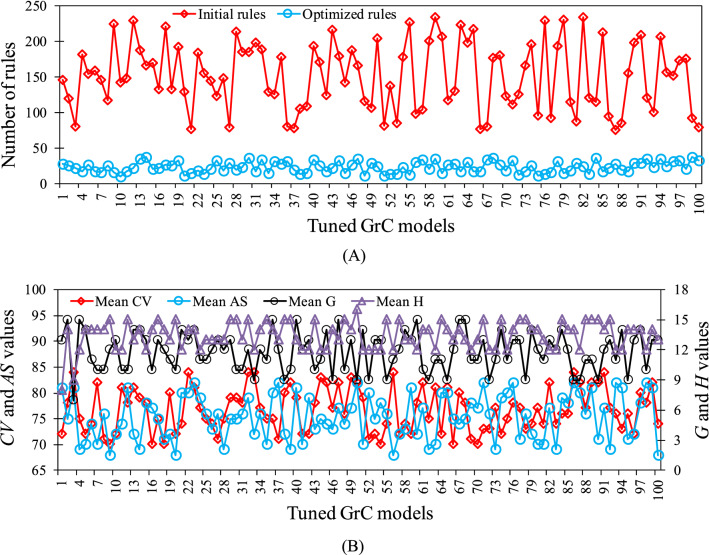
(**A**) Number of initial and optimized rules, and (**B**) the mean values of quality indices for the final rules for each tuned GrC models.

To examine the GrC-ANN model, the database with 503 observations from natural streams and laboratory flumes were scaled between 0 and 1. 40 data instances were selected from the global tracer database for the model verification. Then, 100 distinct training patterns were randomly resampled from the remaining database, i.e. 463 observations, with replacement to tune 100 different ${D}_{x}/H{U}^{*}$ GrC-ANN models. Each training pattern consists of 80 data, and the 40 pre-assigned verification data. The model inputs include, aspect ratio and friction term, and dimensionless target ${D}_{x}/H{U}^{*}$ were clustered based on their indiscernibility in the given attributes. To form the final rule network, GrC-based rule extraction algorithm was used to select the best granules of information by considering the *CE*, *AS*, *G*, and *CV* measures computed for each rule. In this regard, *AS* and *CE* indices were employed to extract the set of possible valid rules by considering minimum and maximum threshold values of 0.75 and 0.5, respectively, in accordance to similar studies in the literature^39,81,82^. At this stage, if a rule caused redundancy in the rule set, it was considered as an active granule and was replaced with a granule that had more consistency in the set of rules. Using the proposed methodology led to extraction of a range of rules, varied from 76 to 234, for tuning the GrC models based on the training patterns (Fig. 5A).

In the next step, the *CV* and *G* indices were applied to prioritize the rules that construct the final rule sets. For the models tuned based on the training patterns, the optimized rules varied from 10 to 38 (Fig. 5A). The mean values of quality indices for the final rules selected for each tuned model are illustrated in Fig. 5B. According to Fig. 5B, the *G* values ranged between 0 and 0.4, indicating the rules’ generality does not pertain to big values of *G*, confirming the results of previous GrC modelling studies^39,74^. The *CV* varied between 0 and 1, pertaining to the numbers of extracted rules by each class and the dataset covered by each rule, following Yao and Yao^74^ findings.

**Figure 6:**
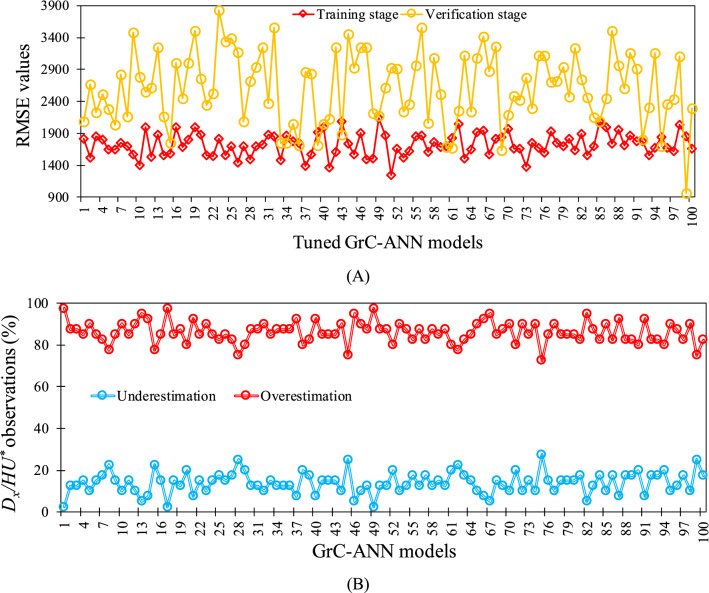
(**A**) Root mean square error (RMSE) values calculated for the tuned ${D}_{x}/H{U}^{*}$ GrC-ANN models in training and verification stages, and (**B**) ${D}_{x}/H{U}^{*}$ observations (%) with underestimation and overestimation in GrC-ANN models tuned by the distinct training patterns.

The 100 optimized rule sets computed correspond to one hundred distinct training patterns, which are then fed to the GrC-ANN modelling structure. In this regard, the rule quality indices were embedded into an ANN structure instead of initial weights, forming a GrC-ANN model corresponding to each optimized rule set. The best network structures describing the relations between the inputs (*W*/*H* and *U*/U^*^) and the output (${D}_{x}/H{U}^{*}$) data were determined based on the quality index of root mean square error (RMSE) for each GrC-ANN model tuned by the distinct training patterns (Fig. 6A). Analysis of the results show the RMSE values for the tuned ${D}_{x}/H{U}^{*}$ GrC-ANN models, in training and verification stages varied from 1251 to 2142 and 966 to 3826, respectively (Fig. 6A).

**Figure 7:**
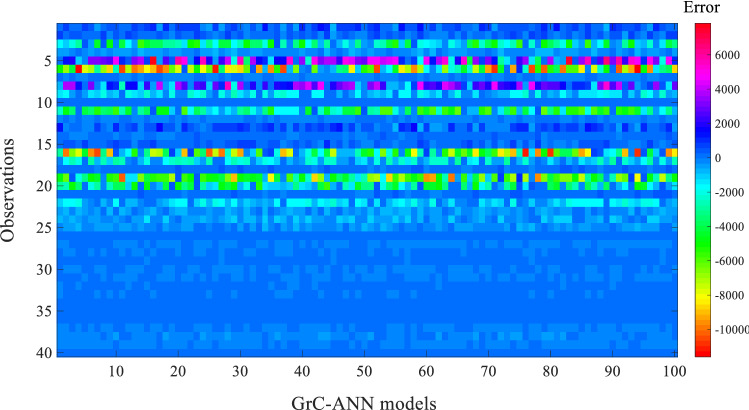
Difference between the true ${D}_{x}/H{U}^{*}$ values and those predicted using GrC-ANN models tuned by the distinct training patterns during the verification stage of the model.

Figure 7 shows the difference between the true (field-estimated) ${D}_{x}/H{U}^{*}$ values and those predicted by each tuned ${D}_{x}/H{U}^{*}$ GrC-ANN model. The minimum (i.e., − 10,934) and the maximum (i.e., 7471) errors were produced by ${D}_{x}/H{U}^{*}$ GrC-ANN models #42 and #100, respectively. In general, the GrC-ANN models overestimate the ${D}_{x}/H{U}^{*}$ values for approximately 86% of the observations (Fig. 6B). Similar overestimation of *D*_*x*_ was reported by Etemad-Shahidi and Taghipour^83^ for the *D*_*x*_ models proposed by Liu^61^, Seo and Cheong^13^, Deng et al.^57^, and Sahay and Dutta^84^. In this study, the overestimation of ${D}_{x}/H{U}^{*}$ could be associated with the RMSE values used as the objective function in the GrC-ANN model. RMSE is a scale-dependent parameter and could lead the model to predict values with lower relative error for large *D*_*x*_ values that rarely exist in the database. In addition, we defined a constraint for the GrC-ANN model to filter out the modeling result for the negative values, which are likely to contribute to the overestimation for small ${D}_{x}/H{U}^{*}$ values that are the dominant feature of the database. However, using the overestimated ${D}_{x}/H{U}^{*}$ values in 1-D ADE models give a lower maximum concentration rate for those locations which are far from the pollutant injection point^12^. Therefore, the tuned ${D}_{x}/H{U}^{*}$ GrC-ANN model must be used with caution in hydro-environmental studies such as outfall design, and risk assessment studies for accidental hazardous pollution.

**Figure 8:**
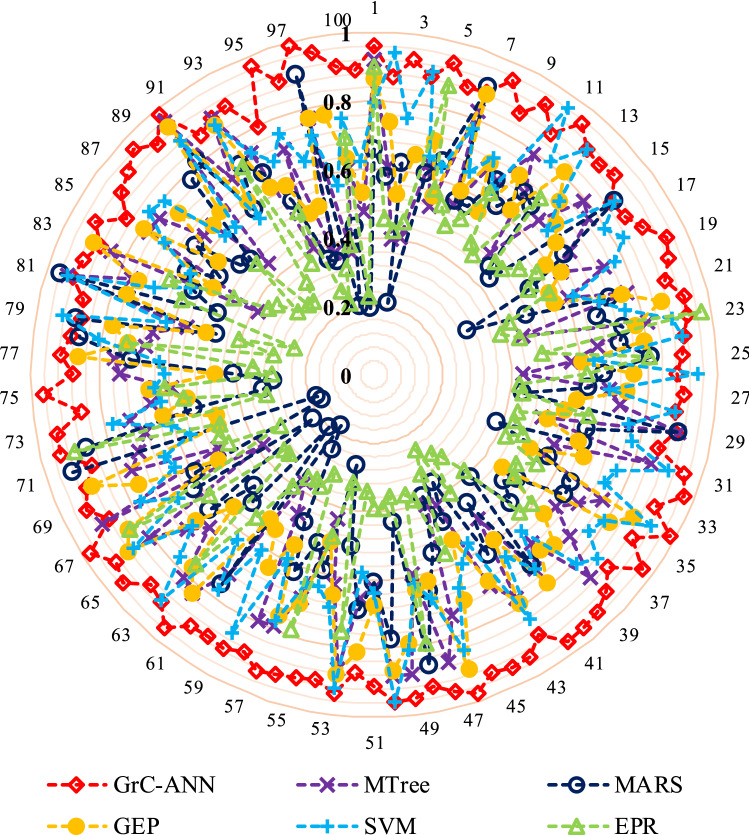
Determination of coefficient (R^2^) values calculated for ${D}_{x}/H{U}^{*}$ prediction during the verification step of the tuned GrC-ANN models developed in this study, and those reported for model tree (MTree), gene-expression programming (GEP), evolutionary polynomial regression (EPR), support vector machine (SVM) and multivariate adaptive regression splines (MARS) by Najafzadeh et al.^85^.

Comparative analysis of the tuned GrC-ANN models developed in this study, and other AI models including model tree (MTree), gene-expression programming (GEP), evolutionary polynomial regression (EPR), support vector machine (SVM1), and multivariate adaptive regression splines (MARS), developed by Najafzadeh et al.^85^, highlights that the proposed GrC-ANN models are capable of better and more robust approximation of longitudinal dispersion (${D}_{x}/H{U}^{*}$) in streams (Fig. 8). Previous studies also confirmed the performance superiority of GrC compared to ANN and adaptive neuro fuzzy inference system (ANFIS) developed for ${D}_{x}/H{U}^{*}$ predictions^43^. As shown in Fig. 8, the determination coefficient (R^2^) values determined for the GrC-ANN models in verification stage, are much larger than those reported for ERP and MARS models. However, the computational cost of GrC-ANN model is more than that for ANN models. In this study, the computational time of GrC-ANN models was approximately 1.8 to 2.6 times greater than that for the ANN models.

### GrC-ANN uncertainty

**Figure 9:**
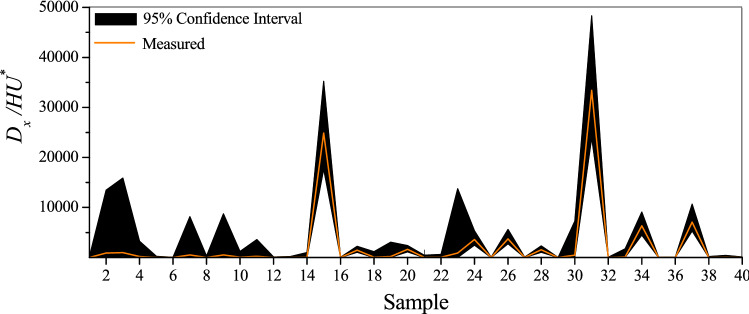
GRC-ANN model uncertainty for estimation of ${D}_{x}/H{U}^{*}$ in streams.

The ${D}_{x}/H{U}^{*}$ values estimated during the verification stage by the 100 GrC-ANN models tuned under distinct training patterns were used to measure the model uncertainty. In this regard, prediction intervals corresponding to each ${D}_{x}/H{U}^{*}$ observation was computed by considering the level of significance of 95% (Fig. 9). These prediction intervals show the deviation from the true ${D}_{x}/H{U}^{*}$ values, denoting the uncertainty associated with the GrC-ANN predictions of longitudinal dispersion in streams.

Figure 9 shows that the true ${D}_{x}/H{U}^{*}$ values are fully located between the lower and upper bands of the uncertainty, concluding the appropriate performance of the GrC-ANN model based on the NDB95PU (%) index. Also the small value of the bandwidth*-factor* (= 0.56) indicates the small deviation of the predicted ${D}_{x}/H{U}^{*}$ values by the GrC-ANN models from the measured values, leading to low uncertainty of the model. Figure 9 shows that the proposed GrC-ANN model has good performance in predicting both large and small ${D}_{x}/H{U}^{*}$ values with a narrow bandwidth of uncertainty, highlighting the model superiority in predicting the ${D}_{x}/H{U}^{*}$ compared to other AI models which are suffering from large uncertainty in estimation of *D*_*x*_^12,42,85^.

**Figure 10:**
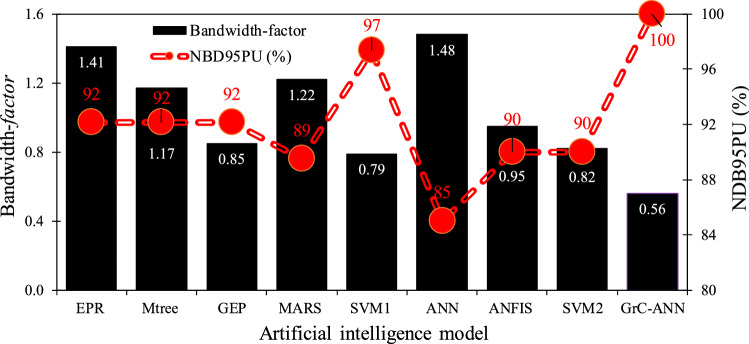
Comparison of the bandwidth-*factor* and the NDB95PU (%) values of the GrC-ANN model developed in this study (i.e., ${D}_{x}/H{U}^{*}$ GrC-ANN), with ANN and ANFIS models^42^, SVM1, GEP, MTree, MARS, and EPR models^85^, and SVM2 model^42^.

However, neither the GrC-ANN model nor other mathematical and statistical models can fully understand and predict the dispersion processes in real streams. Therefore, the results illustrated in Fig. 9 still contain some degree of uncertainty in the prediction of ${D}_{x}/H{U}^{*}$ from GrC-ANN model. To compare the uncertainty of the predicted ${D}_{x}/H{U}^{*}$ from GrC-ANN with other AI models, the bandwidth-*factor* and NDB95PU (%) values computed for these models are illustrated in Fig. 10. This figure shows that ${D}_{x}/H{U}^{*}$ GrC-ANN model has the smallest bandwidth-*factor* value amongst the nine AI-based models examined in this study. Also, ${D}_{x}/H{U}^{*}$ GrC-ANN model has the largest NDB95PU (%) value compared to other AI models (i.e., EPR, MTree, GEP, SVM, MARS, ANN, and ANFIS). These measures suggest that the uncertainty in the prediction of ${D}_{x}/H{U}^{*}$ from GrC-ANN model is far less than those reported for other well-established AI models for the case of pollutant transport in streams.

However, study of the Fig. 9 reveals that despite modified and enhanced training patterns adopted in this study, there remains some uncertainty in the prediction of the ${D}_{x}/H{U}^{*}$ from GrC-ANN model, which can be considerable at times and leading to a wide confidence interval band for some samples. In fact, in the ${D}_{x}/H{U}^{*}$ GrC-ANN modelling process, some rules are eliminated due to low criteria values (i.e., *G*, *AS*, *CV*, and *CE*). Therefore, the selected rules, which govern the final prediction of the model, do not fully represent the complex mechanisms of the longitudinal dispersion in streams, leading to inevitable uncertainty in the predictions by GrC-ANN model. In addition, diversity of streams and the irregularities in geometric characteristics and nonlinearity of the flow hydrodynamics add to the complexity of the mixing mechanisms in the streams. Therefore, full identification, quantification and inclusion of these intricate natural processes in a mathematical or statistical model is not possible. This is correct even for the non-simplified models for prediction of *D*_*x*_, i.e. Equation (2), where estimated *D*_*x*_ values are still not in full agreement with those values measured in the field. For example, the minimum error between the estimated and field-measurement of *D*_*x*_ values occurs for the case of a uniform flow, that is usually less than 30%^68^. In the case of non-uniform flow in large meandrous streams with severe irregularities in bathymetry, and spatiotemporal variations in flow hydrodynamics, the estimated *D*_*x*_ using Eq. (2) largely deviates from the true values^11^. The problem of inaccuracy in modelling predictions raises up when using Eq. (3), derived based on simplified assumptions for Eq. (2), and by exclusion of important parameters influencing *D*_*x*_ such as *S*_*f*_ and *S*_*n*_^5,11,16,86–88^. These excluded parameters are seldom monitored in natural streams due to the difficulties associated with their measurement. Another factor that contribute to the uncertainty in prediction of longitudinal dispersion from GrC-ANN model is the rare presence of very large *D*_*x*_ values in the dataset used in this study. Analysis of the dataset used in this study shows that only around 1% of the 503 global dataset of tracer experiments consists of *D*_*x*_ > 1000 m^2^/s, whilst the maximum value of *D*_*x*_ in the dataset is around 1800 m^2^/s^12^. This absence of very large *D*_*x*_ in the dataset, is leading to uncertainty in the ${D}_{x}/H{U}^{*}$ predicted by the GrC-ANN model.

## Conclusions

Longitudinal dispersion coefficient (*D*_*x*_) influences the transport and fate of pollutants in streams. Given the high spatiotemporal variability of *D*_*x*_, previous AI models with single training pattern cannot capture the uncertainty associated with the predictive models for *D*_*x*_ in streams. This study provides rigorous methodological approach to examine and quantify the uncertainty in the prediction of ${D}_{x}/H{U}^{*}$ from the proposed GrC-ANN model. The detailed analysis of the results highlights that although ${D}_{x}/H{U}^{*}$ predicted by GrC-ANN model outperforms other AI-based dispersion models, there remains some uncertainty in the predicted *D*_*x*_ from the model which need careful consideration and evaluation. This finding suggests that river water quality assessments and environmental management studies should consider the impacts of uncertainty associated with the *D*_*x*_ estimation on the pollutant concentrations, that could result in detrimental impacts on aquatic biodiversity, and ecosystem function in streams as well as the public health. Enhanced data on the flow hydrodynamics and the geometric features in streams (e.g., stream sinuosity and bed shape factor) for the *D*_*x*_ models can further reduce the uncertainty in estimation of longitudinal dispersion parameter.

## Data Availability

The data used in this study can be obtained from https://doi.org/10.1007/s11269-018-2139-6.
